# Perioperative Outcomes After Radical Nephrectomy with Inferior Vena Cava Thrombectomy

**DOI:** 10.3390/cancers17071083

**Published:** 2025-03-24

**Authors:** Nikolaos Pyrgidis, Gerald Bastian Schulz, Christian G. Stief, Iulia Blajan, Troya Ivanova, Annabel Graser, Michael Staehler

**Affiliations:** Department of Urology, University Hospital, Ludwig-Maximilian University, 81377 Munich, Germany; gerald.schulz@med.uni-muenchen.de (G.B.S.); christian.stief@med.uni-muenchen.de (C.G.S.); iulia.blajan@med.uni-muenchen.de (I.B.); troya.ivanova@med.uni-muenchen.de (T.I.); annabel.graser@med.uni-muenchen.de (A.G.); michael.staehler@med.uni-muenchen.de (M.S.)

**Keywords:** cohort study, radical nephrectomy, inferior vena cava thrombectomy, perioperative outcomes, mortality

## Abstract

Background and Objective: We aimed to evaluate current trends and complications after radical nephrectomy with inferior vena cava (IVC) thrombectomy and to provide evidence on the role of the annual hospital caseload on perioperative outcomes. Methods: We used the GeRmAn Nationwide inpatient Data (GRAND), provided by the German Bureau of Statistics (2005–2022). All hospitals performing radical nephrectomy with IVC thrombectomy were subclassified based on their annual caseload to low- (<3 cases/year), intermediate- (3–9 cases/year), and high-volume centers (≥10 cases/year). We included 3608 patients. Key Findings and Limitations: Overall, 1880 (52%) patients underwent surgery in low-, 1466 (40%) in intermediate-, and 848 (8%) in high-volume centers. Most patients (3574, 99%) underwent open surgery. The number of patients undergoing radical nephrectomy with IVC thrombectomy has decreased in the last years. Patients undergoing surgery in low-, intermediate- and high-volume centers had similar baseline characteristics. Operation in high-volume centers, compared to low-volume centers, was associated with lower odds of intensive care unit admission (29% versus 45%, OR: 0.5, 95% CI: 0.4–0.7, *p* < 0.001) and a shorter hospital stay by 3.9 days (95% CI: 2.2–5.6, *p* < 0.001). Importantly, for every additional case performed annually, hospitals improve their perioperative outcomes in terms of mortality (*p* = 0.032), intensive care unit admissions (*p* = 0.002), acute kidney disease (*p* = 0.029), and length of hospital stay (*p* < 0.001). Conclusions and Clinical Implications: The present real-world data demonstrate that, for every additional case performed annually, hospitals improve their perioperative outcomes in terms of major perioperative complications.

## Simple Summary

Patients with kidney cancer that extends into the inferior vena cava often require radical nephrectomy with inferior vena cava thrombectomy. However, outcomes after this procedure may vary depending on the hospital’s experience. This study analyzed national data from Germany to assess how hospital caseload influences surgical complications, intensive care unit admissions, length of hospital stay, and mortality rates. The findings suggest that hospitals performing more of these procedures annually tend to have better patient outcomes, with fewer complications and shorter hospital stays. Despite these benefits, most surgeries in Germany still take place in low-volume centers, highlighting the need for potential centralization of care to improve patient safety and recovery.

## 1. Introduction

Renal cell carcinoma (RCC) is commonly diagnosed in early stages due to the wide implementation of abdominal imaging [1]. Still, about 10% of all patients with RCC are initially diagnosed at a locally advanced stage, including tumors with a thrombus extending into the inferior vena cava (IVC) [2]. As with any non-metastatic RCC, radical nephrectomy with concomitant IVC thrombectomy is the recommended treatment of choice [3]. Compared to smaller, locally confined RCCs, this procedure is associated with higher rates of perioperative morbidity and mortality [4]. Despite advancements in surgical techniques and perioperative management, the complexity of the procedure continues to pose challenges in terms of patient safety and recovery [5].

Recent studies suggest that hospital experience and institutional expertise play a crucial role in mitigating these risks, yet there is no standardized threshold for defining a high-volume center in this setting [6]. Hospitals with higher annual caseload presumably are more likely to provide the required infrastructure and expertise to reduce intraoperative and postoperative complications after radical nephrectomy with IVC thrombectomy [7]. Apparently, higher hospital caseload, rather than higher surgeon volumes, are associated with better perioperative outcomes for other complicated major urological cancer surgeries, such as radical cystectomy [8]. Although similar trends have been observed in bladder and prostate cancer surgeries, data on the impact of hospital caseload on nephrectomy with IVC thrombectomy outcomes remain scarce [9,10]. Understanding these trends could help to optimize patient referrals and improve perioperative care strategies.

Given the paucity of available studies assessing the role of annual hospital caseload on perioperative outcomes, relevant guideline recommendations are missing [11]. Within this scope, we aimed to evaluate the current trends and complications after radical nephrectomy with IVC thrombectomy, as well as to provide evidence about the role of the annual hospital caseload on perioperative outcomes through the largest study in the field.

## 2. Methods

### 2.1. Data Source

For the present analysis, we used the GeRmAn Nationwide inpatient Data (GRAND) from the Federal Bureau of Statistics in Germany. The GRAND study contains all in-hospital patient data from 2005 to 2022 in Germany. Military, psychiatric, and forensic cases are excluded from GRAND. These data are stored anonymized at the Research Data Center of the Federal Bureau of Statistics and access for further analysis was gained upon agreement (LMU-4710-2022). The GRAND contains information on coexisting conditions, surgical procedures, and perioperative outcomes. This information is coded precisely based on the International Statistical Classification of Diseases and Related Health Problems, 10th revision, German modification (ICD-10-GM), and the German Procedure Classification (OPS). The coding guidelines are provided by the German Institute for Medical Documentation and Information and ensure uniform documentation throughout Germany. After the implementation of a diagnosis- and procedure-related remuneration system in Germany (German Diagnosis Related Groups—DRG) in 2004, all hospitals transfer this information to the Institute for the Hospital Remuneration System to receive their remuneration.

### 2.2. Selection Criteria, Coding, and Annual Hospital Caseload Threshold

We included all patients undergoing radical nephrectomy (OPS code: 5-554.4, 5-554.a) with IVC thrombectomy (OPS code: 5-380.97). To obtain patient data on further coexisting conditions, surgical procedures, and perioperative outcomes, we used the available diagnostic and procedural codes (ICD-10-GM and OPS). The primary outcome of the present analysis was to assess the role of the annual hospital caseload on 30-day mortality in patients undergoing radical nephrectomy with IVC thrombectomy. Secondary outcomes included the role of annual hospital caseload on perioperative complications (intensive care unit (ICU) admission, transfusion, sepsis, acute kidney disease, acute embolism, and ileus) and length of hospital stay. Subsequently, we assessed the surgical trends in radical nephrectomy with IVC thrombectomy over recent years in Germany.

### 2.3. Data Synthesis and Statistical Analysis

Due to data anonymity, our research team was not allowed to gain direct access to the patient-level data. As a result, all statistics were performed on our behalf by the Research Data Center of the Federal Bureau of Statistics based on R codes developed by our research team (source: Research Data Center of the Federal Bureau of Statistics, DRG Statistics 2005–2022, own calculations). Subsequently, the summary results were sent to our research group for further evaluation. Approval by an ethics committee or patient informed consent was not necessary according to German legislation.

All hospitals performing radical nephrectomy with IVC thrombectomy were identified through their postal code. Given that there are no recommended thresholds on the annual hospital caseload for radical nephrectomy with IVC thrombectomy, we calculated them based on the Youden’s index with receiver operating characteristic (ROC) analyses. In particular, the Youden’s index was estimated for each point of the ROC curve and its maximum value was used as a criterion to calculate the optimal threshold for annual hospital caseload and 30-day mortality, embolism, admission to the intensive care unit, transfusion, sepsis, and length of hospital stay. Subsequently, the specificity, sensitivity, positive predictive value, and negative predictive value of the annual hospital caseload threshold in optimizing each perioperative outcome were also provided. The corresponding comparisons among low- versus intermediate- versus high-volume centers were performed using chi-squared and Kruskal–Wallis tests. All continuous variables were calculated as medians with interquartile ranges (IQRs), and all categorical variables as frequencies with proportions.

We undertook a multivariable logistic and linear regression analysis to evaluate the effect of the annual hospital caseload on perioperative outcomes (30-day mortality, perioperative complications, and length of hospital stay). The annual hospital caseload was also assessed as a continuous variable. All regression models were adjusted for sex, obesity, age, hypertension, diabetes, history of chronic obstructive pulmonary disease, chronic kidney disease, cerebrovascular accident, chronic heart failure, as well as for the surgical approach and the year of operation. The log-rank test with Kaplan–Meier curves was also used to assess the role of the annual hospital caseload on 30-day mortality. Odds ratios (ORs) with a 95% confidence interval (CI) were estimated for all logistic models, and two-sided *p*-values < 0.05 were considered statistically significant [12].

## 3. Results

### 3.1. Baseline Characteristics

The optimal annual hospital threshold for radical nephrectomy with IVC thrombectomy that may reduce 30-day in-hospital mortality was estimated at 8 cases/year and displayed a sensitivity of 62%, specificity of 49%, positive predictive value of 50%, and negative predictive value of 96%. The optimal annual hospital RC threshold was 2 cases/year for embolism, 3 cases/year for admission to the ICU, 4 cases/year for transfusion, 2 cases/year for sepsis, and 7 cases/year for hospital stay (Table 1). Based on the previous notion, the hospitals performing radical nephrectomy with IVC thrombectomy were subclassified based on their annual caseload to low-volume centers (<3 cases/year), intermediate-volume centers (3–9 cases/year), and high-volume centers (≥10 cases/year).

**Figure 1:**
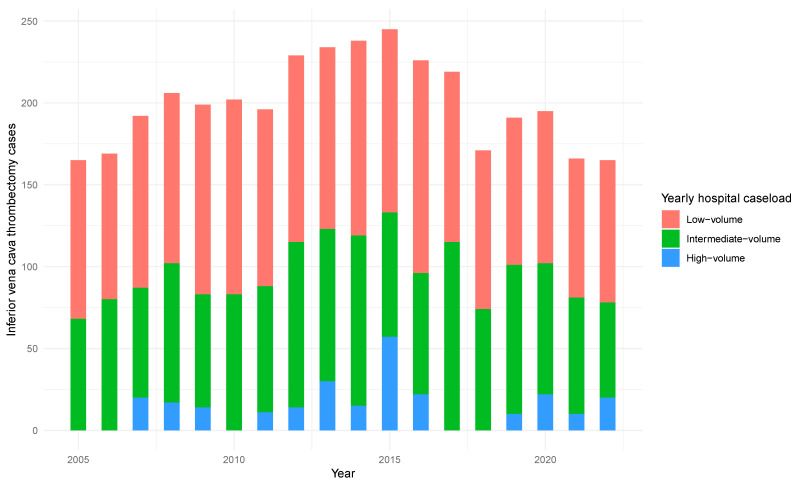
The annual trends for radical nephrectomy with inferior vena cava thrombectomy based on hospital caseload.

A total of 3608 patients with a median age of 69 years (IQR: 60–76) underwent radical nephrectomy with IVC thrombectomy in Germany from 2005 to 2022. Of them, 1880 (52%) underwent surgery in low-, 1466 (40%) in intermediate-, and 848 (8%) in high-volume centers. Overall, 2337 (65%) patients were male, 2179 (60%) had hypertension, 859 (24%) had chronic kidney disease, and 846 (23%) had diabetes. Almost all patients (3574, 99%) underwent an open surgical approach. Patients undergoing surgery in low-, intermediate-, and high-volume centers had similar baseline characteristics (Table 2). The number of patients undergoing radical nephrectomy with IVC thrombectomy has decreased over recent years and was not affected by the COVID-19 pandemic. The annual trends for radical nephrectomy with IVC thrombectomy are presented in Figure 1.

### 3.2. Effect of Surgery on 30-Day Perioperative Mortality

**Figure 2:**
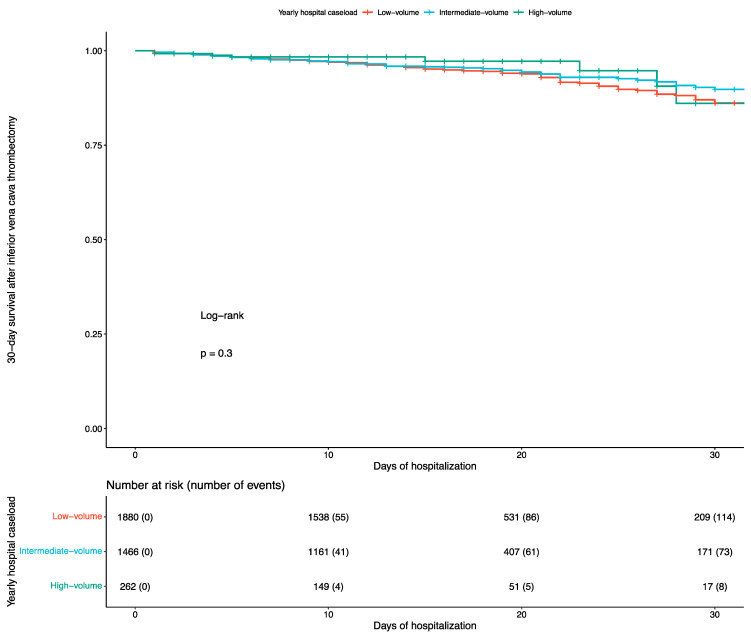
Kaplan–Meier curve for 30-day survival in patients undergoing radical nephrectomy with inferior vena cava thrombectomy based on hospital caseload.

After radical nephrectomy with IVC thrombectomy, the 30-day in-hospital mortality rate was 5.4% (195 deaths), and the overall in-hospital mortality rate was 6.7% (241 deaths). A total of 114 (6.1%) 30-day deaths occurred in low-, 73 (5%) in intermediate-, and 8 (3.1%) in high-volume centers. In the multivariate analysis, after adjusting for major risk factors (Table 3), intermediate- and high-volume centers had similar in-hospital 30-day mortality rates compared to low-volume centers (OR: 0.8, 95% CI: 0.6 to 1.2, *p* = 0.4 and OR: 0.5, 95% CI: 0.2 to 1, *p* = 0.07, respectively). This was also observed in the time-to-death analysis (log-rank test: *p* = 0.3). The corresponding Kaplan–Meier curve is depicted in Figure 2. Still, for every additional annual hospital case, mortality decreased by 5% (OR: 0.95, 95% CI: 0.9 to 0.99, *p* = 0.032).

### 3.3. Effect of Surgery on Perioperative Complications

Overall, 1705 (47%) patients required ICU admission. A total of 845 (45%) ICU admissions occurred in low-, 783 (53%) in intermediate-, and 77 (29%) in high-volume centers. Surgery in intermediate-volume centers, compared to low-volume centers, was associated with higher odds of ICU admission (OR: 1.4, 95% CI: 1.3 to 1.7, *p* < 0.001), whereas surgery in high-volume centers, compared to low-volume centers, was associated with lower odds of ICU admission (OR: 0.5, 95% CI: 0.4 to 0.7, *p* < 0.001). Still, for every additional annual hospital case, ICU admissions decreased by 3% (OR: 0.97, 95% CI: 0.96 to 0.99, *p* = 0.002).

A total of 2598 (72%) patients required perioperative transfusion (1327 (71%) in low-, 1077 (73%) in intermediate-, and 194 (74%) in high-volume centers). Operation in intermediate-volume centers, compared to low-volume centers, was associated with higher odds of transfusion (OR: 1.2, 95% CI: 1 to 1.4, *p* = 0.04). Moreover, perioperative acute kidney disease occurred in 488 (14%) patients, sepsis in 136 (3.8%), acute embolism in 364 (10%), and ileus in 125 (3.5%). No statistically significant differences in cardiovascular complications were observed among low-, intermediate-, or high-volume centers. Nevertheless, for every additional annual hospital case, acute kidney disease decreased by 3% (OR: 0.97, 95% CI: 0.94 to 1, *p* = 0.029). The median length of hospital stay after surgery was 14 (IQR: 10–20) days. Surgery in low-, compared to high-volume centers, was associated with a longer hospital stay by 3.9 days (95% CI: 2.2 to 5.6, *p* < 0.001). Accordingly, for every additional annual hospital case, the length of hospital stay decreased by 0.28 days (95% CI: 0.16 to 0.39, *p* < 0.001). The multivariable analyses and the total number of events in each group are presented in Table 3.

## 4. Discussion

Due to the lack of available evidence on the matter, we established an optimal annual hospital caseload based on sophisticated statistical methods. Nevertheless, this recommended annual hospital caseload threshold for radical nephrectomy with IVC thrombectomy resulted in contradictory findings. In particular, after adjusting for major risk factors, patients undergoing surgery in high-volume centers were discharged earlier from the hospital and were admitted less frequently to the ICU. On the contrary, surgery at intermediate-volume centers was associated with more ICU admissions and higher odds of transfusions compared to low-volume centers. Moreover, mortality rates and further major perioperative complications did not differ between low-, intermediate-, and high-volume centers. Thus, we also evaluated the effect of annual hospital caseload as a continuous variable. Our findings indicate that, for every additional case performed annually, hospitals improve their perioperative outcomes in terms of mortality, ICU admissions, acute kidney disease, and length of hospital stay. Despite these findings, it seems that there is no centralization in the management of radical nephrectomy with IVC thrombectomy in Germany, since the number of patients undergoing surgery in high-volume centers is low and has not increased over recent years. Of note, the total number of patients undergoing radical nephrectomy with IVC thrombectomy has decreased over recent years.

Although it has been repeatedly shown that higher hospital and surgeon caseloads correlate with better perioperative outcomes for other complicated oncological procedures, studies on radical nephrectomy with IVC thrombectomy are scarce and present contradictory findings [13,14]. Even though a study from the USA National Cancer Data Base suggested that surgery at high-volume centers is associated with a 24% risk reduction for long-term mortality compared to low-volume centers, the authors concluded that surgery at a high-volume center does not reduce perioperative mortality [15]. Moreover, the authors did not explore the effect of annual hospital caseload on perioperative morbidity and further studies on the matter are lacking. To complicate things further, the authors defined the thresholds of annual hospital caseload based on percentiles, rendering the interpretation of their findings problematic. In particular, low-volume hospitals were classified as those between the 0 and 75th percentile (<0.67 annual cases), intermediate-volume hospitals as those between the 75th and 95th percentile (0.67–2.99 annual cases), and high-volume hospitals as those above the 95th percentile (>3 annual cases) [15]. Furthermore, Toren et al., using a Canadian population-based database, suggested that increasing surgeon and hospital volumes does not correlate with lower in-hospital mortality and morbidity in multivariate regression analyses [16]. Accordingly, Yap et al., using the Ontario Cancer Registry, reported 30-day mortality rates of 2.8%. Nevertheless, 80% of the included patients presented with a thrombus in the renal vein. The authors concluded that surgeons who performed at least two cases per year presented better 30-day mortality compared to surgeons who performed one case per year. Still, increased hospital caseload was not associated with better mortality outcomes. Accordingly, no analyses on perioperative complications were performed [17].

Although a plethora of existing studies have showcased the potential benefits of centralization for complicated cancer care [18], the findings of our study, as well as those of previous studies, indicate that the management of patients with RCC and IVC thrombus remains decentralized. In particular, most surgeries in the USA, Canada, and Germany are performed in centers that perform less than three cases annually [15,16,17]. Accordingly, high-volume, single-center cohort studies on the matter are lacking [19,20,21]. Of note, meta-analyses indicate that robotic radical nephrectomy with IVC thrombectomy is associated with lower complication rates, less blood loss, and a shorter length of hospital stay compared to open surgery [22,23,24]. The overall complication rate of robotic radical nephrectomy with IVC thrombectomy is about 15%, compared to 37% after open surgery [22]. Accordingly, major complication rates are 6.6% after a robot-assisted approach, compared to 18% after open surgery [22]. The overall 30-day postoperative mortality is approximately 2% [25]. Overall, it seems that robotic radical nephrectomy with IVC thrombectomy is feasible and safe in experienced hands [26]. Nevertheless, it should be highlighted that, in Germany, almost all patients still undergo open surgery and that there is a high risk of selection bias in published studies. Still, recent data from Germany indicate the superiority of robot-assisted radical and partial nephrectomy [27].

Although we report, to the best of our knowledge, the first study on the trends and perioperative outcomes after radical nephrectomy with IVC thrombectomy based on the annual hospital caseload, it should be highlighted that our findings are not devoid of some limitations, which need to be considered. Firstly, our analyses derive from retrospective, administrative, billing data, and are thus prone to coding errors and misclassifications. Although these data are accurate and are regularly assessed by independent medical task forces from healthcare insurances, important information is not collected. In particular, the tumor size and location, the extent of IVC thrombus, the patient’s laboratory findings, the operative time, and the oncological status (histological findings, TNM classification, and surgical margins) are not available in the GRAND study. Based on the previous notion, the variety of patient-, tumor-, and provider-related characteristics that are missing might have introduced selection bias in the present analysis. Furthermore, data on mortality and morbidity after hospital discharge, readmission, reoperation, or embolization rates, as well as functional outcomes, and follow-up data are not collected in the GRAND study. Importantly, intraoperative complications could not be separated from postoperative complications, and we could not use a standardized approach for complications, such as the Clavien–Dindo classification. Our analyses were also limited to hospital caseload rather than surgeon caseload. Still, since a multidisciplinary approach is needed in such operations, it seems that hospital caseload is the appropriate surrogate. Similarly, the effect of the teaching status of the institution on perioperative outcomes was not evaluated [28]. Moreover, it was beyond the scope of the present study to assess outcomes after the removal of the thrombus in the renal vein. Accordingly, due to the low number of patients undergoing minimally invasive surgery, we did not compare outcomes based on the surgical approach. Finally, our analyses are restricted to data from Germany and, therefore, cannot be extrapolated to other healthcare systems.

## 5. Conclusions

The present nationwide, real-world data from Germany demonstrate that, for every additional case of radical nephrectomy with IVC thrombectomy performed annually, hospitals improve their perioperative outcomes in terms of mortality, ICU admissions, acute kidney disease, and length of hospital stay. However, the total number of patients undergoing radical nephrectomy with IVC thrombectomy in high-volume centers is still low. Accordingly, minimally invasive surgery for radical nephrectomy with IVC thrombectomy is not yet widely implemented across German Urology departments. Based on the previous notion, most patients undergo surgery in low-volume centers, suggesting that a plethora of Urology departments prefer to perform radical nephrectomy with IVC thrombectomy in Germany and not to further refer patients to centers with higher hospital caseload volumes.

## Figures and Tables

**Table 1:** Annual hospital caseload threshold for important perioperative complications after radical nephrectomy with inferior vena cava thrombectomy, based on the maximum Youden’s index of the ROC analysis. ICU: intensive care unit, NPV: negative predictive value, PPV: positive predictive value.

Complication	Hospital Caseload Threshold	Specificity	Sensitivity	NPV	PPV
30-day mortality	8	62%	49%	96%	50%
Embolism	2	33%	71%	91%	10%
Admission to the ICU	3	54%	51%	55%	50%
Transfusion	4	70%	34%	29%	74%
Sepsis	2	32%	73%	97%	4%
Hospital stay	7	79%	65%	50%	88%

**Table 2:** Baseline characteristics of the included patients undergoing radical nephrectomy with inferior vena cava thrombectomy based on the annual hospital caseload. The variables are presented as medians with interquartile ranges or frequencies with proportions. The Kruskal–Wallis test was performed for comparisons between continuous variables and the chi-squared test for comparisons between categorical variables. The bold cells indicate statistically significant *p*-values.

Characteristic	Overall, *n* = 3608	Low Volume (<3), *n* = 1880	Intermediate Volume (3–9), *n* = 1466	High Volume (≥10), *n* = 262	*p*-Value
Age (years)	69 (60–76)	69 (61–76)	68 (59–75)	69 (61–76)	**0.003**
Males	2337 (65%)	1221 (65%)	945 (64%)	171 (65%)	0.94
Diabetes	846 (23%)	440 (23%)	352 (24%)	54 (21%)	0.49
Chronic heart failure	353 (9.8%)	205 (11%)	128 (8.7%)	20 (7.6%)	0.053
Chronic obstructive pulmonary disease	258 (7.2%)	139 (7.4%)	106 (7.2%)	13 (5.0%)	0.35
Chronic kidney disease	859 (24%)	468 (25%)	329 (22%)	62 (24%)	0.26
Cerebrovascular disease	115 (3.2%)	63 (3.4%)	47 (3.2%)	5 (1.9%)	0.46
Dementia	36 (1.0%)	24 (1.3%)	12 (0.8%)	0 (0%)	0.10
Hypertension	2179 (60%)	1168 (62%)	856 (58%)	155 (59%)	0.083
Obesity	356 (9.9%)	182 (9.7%)	147 (10%)	27 (10%)	0.92
Operative technique					0.31
Open	3574 (99%)	1857 (99%)	1455 (99%)	262 (100%)	
Laparoscopic	20 (0.6%)	13 (0.7%)	7 (0.5%)	0 (0%)	
Robotic	14 (0.4%)	10 (0.5%)	4 (0.3%)	0 (0%)	
Year of surgery					
2005	165 (4.6%)	97 (5.2%)	68 (4.6%)	0 (0%)	
2006	169 (4.7%)	89 (4.7%)	80 (5.5%)	0 (0%)	
2007	192 (5.3%)	105 (5.6%)	67 (4.6%)	20 (7.6%)	
2008	206 (5.7%)	104 (5.5%)	85 (5.8%)	17 (6.5%)	
2009	199 (5.5%)	116 (6.2%)	69 (4.7%)	14 (5.3%)	
2010	202 (5.6%)	119 (6.3%)	83 (5.7%)	0 (0%)	
2011	196 (5.4%)	108 (5.7%)	77 (5.3%)	11 (4.2%)	
2012	229 (6.3%)	114 (6.1%)	101 (6.9%)	14 (5.3%)	
2013	234 (6.5%)	111 (5.9%)	93 (6.3%)	30 (11%)	
2014	238 (6.6%)	119 (6.3%)	104 (7.1%)	15 (5.7%)	
2015	245 (6.8%)	112 (6.0%)	76 (5.2%)	57 (22%)	
2016	226 (6.3%)	130 (6.9%)	74 (5.0%)	22 (8.4%)	
2017	219 (6.1%)	104 (5.5%)	115 (7.8%)	0 (0%)	
2018	171 (4.7%)	97 (5.2%)	74 (5.0%)	0 (0%)	
2019	191 (5.3%)	90 (4.8%)	91 (6.2%)	10 (3.8%)	
2020	195 (5.4%)	93 (4.9%)	80 (5.5%)	22 (8.4%)	
2021	166 (4.6%)	85 (4.5%)	71 (4.8%)	10 (3.8%)	
2022	165 (4.6%)	87 (4.6%)	58 (4.0%)	20 (7.6%)	

**Table 3:** Multivariable logistic regression analysis of the effect of the annual hospital caseload for radical nephrectomy with inferior vena cava thrombectomy on major perioperative outcomes. All models are adjusted for sex, age, obesity, history of chronic obstructive pulmonary disease, chronic heart failure, chronic kidney disease, cerebrovascular accident, hypertension, diabetes, surgical approach, and year of operation. The bold cells indicate statistically significant *p*-values. ICU: intensive care unit, OR: odds ratio.

Outcome	Low Volume (<3)		Intermediate Volume (3–9)		High Volume (≥10)		Increase in Annual Hospital Caseload
	Cases	Estimate (95% CI), *p*-Value	Cases	Estimate (95% CI), *p*-Value	Cases	Estimate (95% CI), *p*-Value	Estimate (95% CI), *p*-Value
30-day mortality	114 (6.1%)	—	73 (5%)	0.8 (0.6, 1.2), 0.4	8 (3.1%)	0.5 (0.2, 1), 0.07	0.95 (0.9, 0.99), **0.032**
ICU admission	845 (45%)	—	783 (53%)	1.4 (1.3, 1.7), **<0.001**	77 (29%)	0.5 (0.4, 0.7), **<0.001**	0.97 (0.96, 0.99), **0.002**
Transfusion	1327 (71%)	—	1077 (73%)	1.2 (1, 1.4), **0.04**	194 (74%)	1.2 (0.9, 1.7), 0.2	1.02 (1, 1.04), 0.052
Sepsis	68 (3.6%)	—	61 (4.2%)	1.2 (0.8, 1.7), 0.4	7 (2.7%)	0.7 (0.3, 1.5), 0.4	0.97 (0.92, 1.02), 0.3
Acute kidney disease	260 (14%)	—	200 (14%)	1 (0.8, 1.3), 0.9	28 (11%)	0.7 (0.4, 1), 0.07	0.97 (0.94, 1), **0.029**
Acute embolism	191 (10%)	—	141 (9.6%)	0.9 (0.7, 1.2), 0.5	32 (12%)	1.2 (0.8, 1.8), 0.3	1 (0.97, 1.03), 0.8
Ileus	73 (3.9%)	—	44 (3%)	0.8 (0.5, 1.1), 0.2	8 (3.1%)	0.7 (0.3, 1.5), 0.4	0.96 (0.9, 1.01), 0.14
Length of hospital stay	14 (11–21)	—	14 (10–20)	−0.1 (−1, 1), 0.8	11 (8–17)	−3.9 (−5.6, −2.2), **<0.001**	−0.28 (−0.39, −0.16), **<0.001**

## Data Availability

Data is unavailable due to privacy or ethical restrictions.
